# Ultrasensitive Visual Detection of HIV DNA Biomarkers via a Multi-amplification Nanoplatform

**DOI:** 10.1038/srep23949

**Published:** 2016-04-01

**Authors:** Yuyin Long, Cuisong Zhou, Congmin Wang, Honglian Cai, Cuiyun Yin, Qiufang Yang, Dan Xiao

**Affiliations:** 1 College of Chemistry, Sichuan University, 29 Wangjiang Road, Chengdu 610064, People’s Republic of China; 2 College of Chemical Engineering, Sichuan University, 29 Wangjiang Road, Chengdu 610065, People’s Republic of China

## Abstract

Methodologies to detect disease biomarkers at ultralow concentrations can potentially improve the standard of living. A facile and label-free multi-amplification strategy is proposed for the ultrasensitive visual detection of HIV DNA biomarkers in real physiological media. This multi-amplification strategy not only exhibits a signficantly low detection limit down to 4.8 pM but also provides a label-free, cost-effective and facile technique for visualizing a few molecules of nucleic acid analyte with the naked eye. Importantly, the biosensor is capable of discriminating single-based mismatch lower than 5.0 nM in human serum samples. Moreover, the visual sensing platform exhibits excellent specificity, acceptable reusability and a long-term stability. All these advantages could be attributed to the nanofibrous sensing platform that 1) has a high surface-area-to-volume provided by electrospun nanofibrous membrane, and 2) combines glucose oxidase (GOx) biocatalysis, DNAzyme-catalyzed colorimetric reaction and catalytic hairpin assembly (CHA) recycling amplification together. This multi-amplification nanoplatform promises label-free and visual single-based mismatch DNA monitoring with high sensitivity and specificity, suggesting wide applications that range from virus detection to genetic disease diagnosis.

Since disease DNA biomarkers are usually present in very small amounts, methodologies for ultrasensitive detection play a crucial role in the fields of clinical diagnosis, gene therapy and virus identification^1^,^2^,^3^. Current strategies often require sophisticated instruments that may not be available in laboratories with fewer resources^4^,^5^. Recently, visual DNA detection has become increasingly important for the laboratory and point-of-care diagnostic applications because of its satisfied sensitivity, feature simplicity and portability^6^,^7^,^8^,^9^. However, it is still desired to develop an ultrasensitive sensor for visualizing disease biomarkers at ultralow concentrations. This issue can be resolved using a colorimetric multi-amplification strategy based on a nanofibrous sensing platform.

The G-quadruplex/hemin complex, acting as an activated horseradish peroxidase (HRP)-mimicking DNAzyme, can catalyze the oxidation of 2,2′-azino-bis(3-ethylbenzthiazoline-6-sulfonic acid) (ABTS^2−^) by H_2_O_2_ to produce a colorimetric signal that can be visualized by the naked eye^8^,^9^,^10^. The visualization strategy based on the DNAzyme has been used to detect some important DNA biomarkers, such as human immunodeficiency virus (HIV) DNA and Streptococcus pneumonia with the detection limit of 2.5 pM and 32 pM, respectively^8^,^9^. Catalytic hairpin assembly (CHA) is an enzyme-free and nucleic acid amplification method^9^,^11^,^12^,^13^. As an isothermal nucleic acid amplification reaction, CHA is much more tractable than the polymerase chain reaction (PCR) for the amplification of nucleic acid analytes in point-of-care applications^11^. Importantly, compared with rolling circle amplification (RCA), hybridization chain reaction (HCR) and strand displacement amplification (SDA), CHA allows a very low false-positive signal by the inhibition of nonspecific or parasitic amplicon^7^,^14^,^15^. For the CHA amplification reaction, briefly, two stable species of DNA hairpins (H1 and H2) coexist in solution until an initiator strand is introduced. A target DNA, used as the initiator, can trigger a cascade of hybridization events to yield H1-H2 double helices. Some groups engineered H2 containing G-enriched DNA fragment within its hairpin part. The G-enriched DNA fragment can be exposed upon CHA amplification reaction, further forming G-quadruplex/hemin DNAzyme that catalyzes a colorimetric reaction^9^,^12^.

Electrospun nanofibrous membranes have significant potential as an attractive sensing interface platform because of their high surface-area-to-volume ratio, porous structure, and effective interaction with analytes^16^,^17^,^18^,^19^,^20^,^21^. Recently, some electrospun nanofibrous membranes have been fabricated as colorimetric sensors for visual detection, including, for example, Cu^2+^, Hg^2+^ and formaldehyde^19^,^20^,^21^. Our group previously reported that glucose oxidases (GOx)-functionalized fibrous membrane exhibited a fast and sensitive luminescence response to trace amount of glucose in blood serum sample. The biosensor has a detection limit of 0.1 nM and a quick response time of 1 second^22^. Compared to a thin film, the fibrous membrane exhibited an improved luminescence response from 15% to 73%^22^. It has been demonstrated that both GOx biocatalysis and intermolecular interaction efficiency were improved owing to the fibrous sensing interface. Also, several electrochemical or ECL signal amplifications are reported through the use of fibrous sensing platforms^23^,^24^,^25^,^26^. Therefore, in addition to GOx biocatalysis, it is supposed that fibrous sensing platform will also improve the CHA amplification and DNAzyme-catalyzed colorimetric reaction. However, to the best of our knowledge, no study reporting on CHA amplification supported by electrospun nanofibrous membrane has been carried out.

In this work, a colorimetric multi-amplification strategy is proposed for ultrasensitive detection of disease DNA biomarker based on the combination of nanofibrous membrane and three different amplification reactions. Here, the model target DNA oligonucleotide sequence is a HIV DNA biomarker. Argon plasma treatment, a surface-modification technique, is used to improve the hydrophilicity of the nanofibrous membrane and to achieve the uniform immobilization of both protein and DNA recognition probe on the nanofibrous surface. Owing to the combined the CHA amplification reaction, the DNAzyme-catalyzed colorimetric reaction and GOx biocatalysis reaction on the nanofibrous surface, a cost-effective, regenerative and visual biosensing platform was developed. The multi-amplification strategy was supposed to exhibit the advantages in sensing trace target DNA and discriminating single-based mismatch at nanomolar level in human serum samples, which could further be extended to develop various ultrasensitive disease biomarker sensors for virus detection, genetic disease diagnosis and clinical analysis.

## Results and Discussion

### Design of the multi-amplification strategy

**Figure 1:**
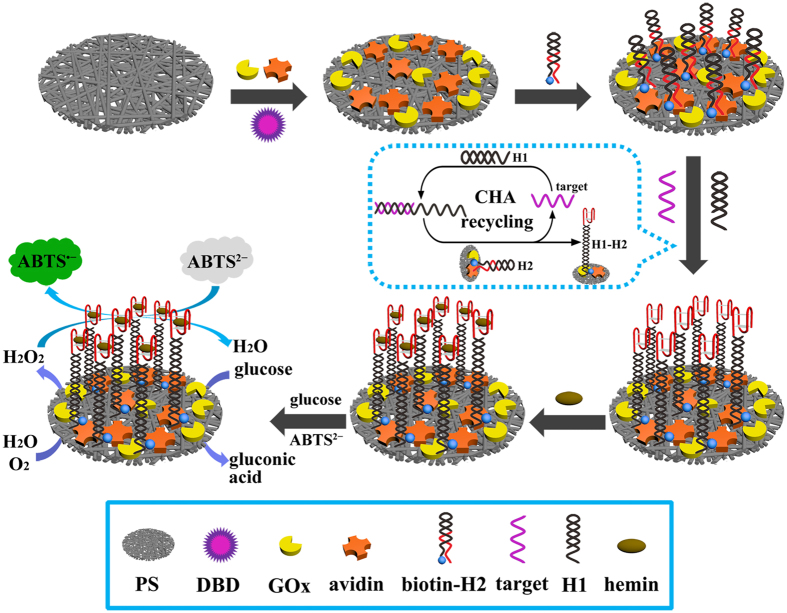
Visual detection of target HIV based on the multi-amplification nanofibrous sensing platform.

A facile and label-free multi-amplification strategy based on a functionalized nanoplatform for ultrasensitive visual detection of HIV DNA biomarker is reported (Fig. 1). The 3–D randomly oriented PS nanofibrous membrane was produced by employing electrostatic repulsions of highly charged PS polymer jets, according to our reported electrospinning technique^22^,^23^,^24^. Based on its advantages of high surface-area-to-volume, porous structure and efficient interaction with analytes, the PS nanofibrous membrane was used as the potential sensing interface material in this work. It has been demonstrated that argon plasma treatment can cause an increase of oxygen-containing groups (mainly –OH and –COOH) in polymer membrane^27^,^28^, thus carrying many negative charges. Therefore, argon plasma treatment was chosen to perform surface modification of the PS nanofibrous membrane. The negatively charged surface of the nanofibrous membrane should be capable of electrostatic interaction with positively charged avidin whose *p*I is 10 in Tris buffer (pH = 7.5). Hence, a uniform and efficient immobilization of avidin on the nanofibrous membrane can be guaranteed.

Multi-amplification reactions include CHA amplification, DNAzyme-catalyzed colorimetric reaction and GOx biocatalysis. As a proof-of-concept, the HIV DNA biomarker was used as the model HIV target. As shown in Fig. 1, biotin-labeled H2 is modified on the nanofibrous membrane surface via strong interaction between avidin and biotin (*Ka* ≈ 10^15^ M^−1^)^29^,^30^. This is followed by CHA amplification reaction on the nanofibrous membrane surface, where the target HIV is used as an initiator to trigger a cascade of hybridization events to yield H1–H2 double helices. Consequently, G-enriched DNA fragment of H2 is exposed to form activated HRP-mimicking DNAzyme upon the binding of G-quadruplex with hemin. In the presence of H_2_O_2_ generated form GOx biocatalysis, the DNAzyme can catalyze the oxidation of colorless ABTS^2−^ to produce a green color of ABTS^−^. The multi-functionalized membrane is named as DNAzyme/GOx/PS nanofibrous membrane. Therefore, a multi-amplification strategy of colorimetric signal can be obtained for the ultrasensitive visual detection of target HIV. Because of its high surface-area-to-volume, improved GOx biocatalysis, and enhanced CHA amplification efficiency, the facile, label-free, ultrasensitive and reusable visual biosensor nanoplatform is expected to have wide applications ranging from virus detection to genetic disease diagnosis.

### Feasibility of the multi-amplification strategy

**Figure 2:**
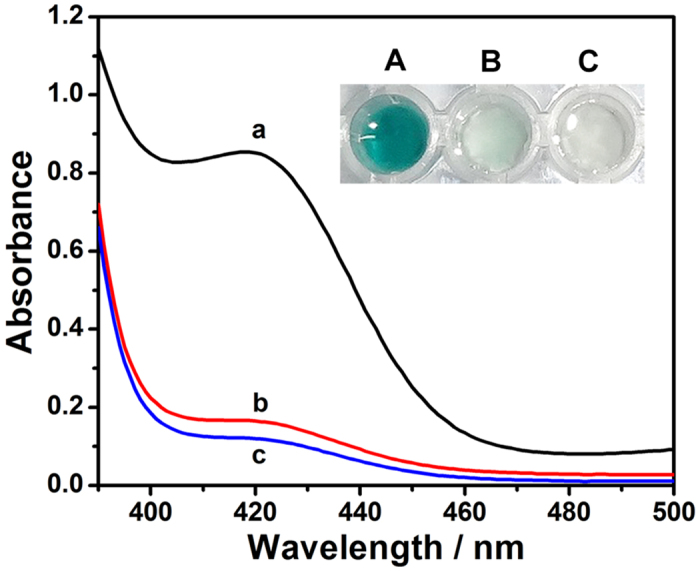
UV–vis absorption spectra of the sensing platform based on nanofibrous membrane under different conditions: (a) biotin-H2 (1.0 μM), H1 (1.0 μM), T_HIV_ (250 nM), hemin (4.0 μM); (b) biotin-H2 (1.0 μM), H1 (1.0 μM), T_HIV_ (0 nM), hemin (4.0 μM); (c) biotin-H2 (1.0 μM), H1 (1.0 μM), T_HIV_ (250 nM), hemin (0 μM). Inset shows corresponding photograph of the color change.

To detect the feasibility of the multi-amplification strategy, it is necessary to ensure that these three amplification reactions occur on the surface of the DNAzyme/GOx/PS nanofibrous membrane. Herein, CHA amplification, DNAzyme-catalyzed colorimetric reaction and GOx biocatalysis reaction were tested one by one. First, in the absence of target HIV, the multi-functionalized nanofibrous membrane did not change the color of the colorimetric solution (Fig. 2B). Also, its corresponding absorbance value was very low (Fig. 2, curve b). However, a strong absorbance signal enhancement was observed at 418 nm after addition of 250 nM target HIV (Fig. 2, curve a). The absorbance value was increased by 414% over that of curve b. In agreement with our expectation, the DNAzyme/GOx/PS nanofibrous membrane changes the colorimetric solution from colorless to deep green (Fig. 2A). It is because the generation of the DNAzyme caused by CHA amplification reaction. The CHA amplification reaction triggered by target HIV was also confirmed using the native polyacrylamide gel electrophoresis (PAGE) data (Figure S1).

To confirm the DNAzyme-catalyzed colorimetric reaction, the multi-functionalized nanofibrous membrane was soaked in the colorimetric solution containing 250 nM target HIV but no hemin. Barely any color change could be observed (Fig. 2C). The corresponding absorbance value was very low (Fig. 2, curve c). Although plenty of G-quadruplex was exposed, as a result of CHA amplification reaction, it is supposed that no DNAzyme was produced in the absence of hemin. The hypothesis was supported by the significant absorbance increase of 600% after the addition of hemin. Correspondingly, the colorimetric solution changed from colorless to deep green in the presence of hemin (Fig. 2A). This change could be ascribed to the formation of the DNAzyme that acts as the key catalyst for the colorimetric system.

Control experiments demonstrated that both avidin and GOx were firmly modified on the surface of the nanofibrous membrane upon the plasma treatment. In contrast to the DNAzyme/GOx/PS nanofibrous membrane, when the DNAzyme/PS nanofibrous membrane without GOx was soaked into the colorimetric solution, no observable color change was captured. Herein, the GOx biocatalysis reaction is confirmed as a key amplification reaction for the visual detection. These results indicated that target HIV can be visually detected using the nanofibrous sensing platform.

### Improved performance of the DNAzyme/GOx/PS nanofibrous membrane for visualization of target HIV

**Figure 3:**
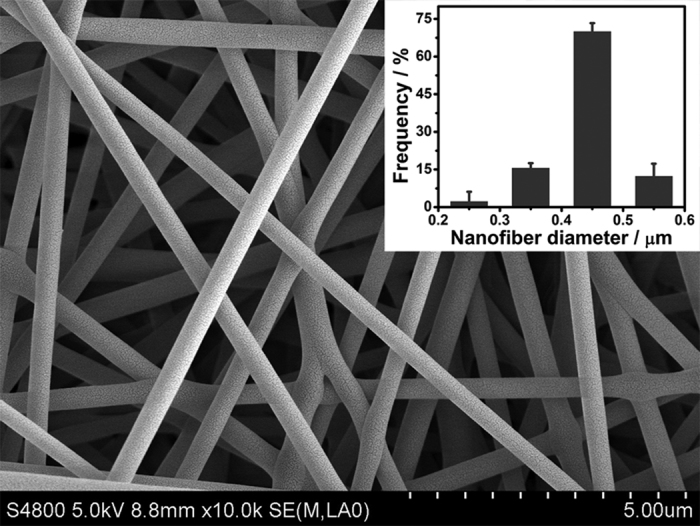
SEM image of DNAzyme/GOx/PS nanofibers. An inset shows the diameter distribution of DNAzyme/GOx/PS nanofibers.

The SEM image shows that DNAzyme/GOx/PS nanofibers with an average diameter of about 430 ± 19 nm were evenly and randomly oriented as porous nanofibrous membrane (Fig. 3). First, the effect of the plasma treatment on surface modification of the nanofibrous membrane was evaluated. The DNAzyme/GOx/PS nanofibrous membrane was fabricated through the use of the plasma-treated nanofibrous membrane followed by a series of functionalization processes (see Experimental Section). After the DNAzyme/GOx/PS nanofibrous membrane was soaked in the colorimetric solution for 20 min, the color of the system changed from colorless to deep green (Figure S2A). In contrast, for the nanofibrous membrane without plasma treatment, followed by the same functionalization process, no observable color change was shown (Figure S2B). Therefore, the plasma treatment was demonstrated as an effective surface modification technique for the nanofibrous membrane. As a result, the plasma-treated nanofibrous membrane can act as an attractive 3–D matrix for carrying biomolecules.

**Figure 4:**
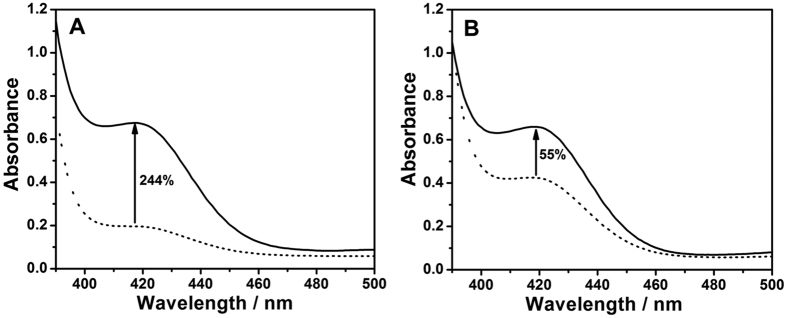
Performance comparison of the DNAzyme/GOx/PS nanofibrous membrane (**A**) with the aqueous solution (**B**) containing 0 nM (dot line) or 5.0 nM (solid line) target HIV in colorimetric solution.

In order to further confirm the improved performance of the multifunctionalized nanofibrous membrane, we compared its colorimetric response that from an aqueous solution (Fig. 4). To guarantee a fair comparison, amounts of reagents in the aqueous solution (Fig. 4B) were the same as those used in Fig. 4A. For the functionalized nanofibrous platform, the addition of 5.0 nM target HIV caused a high absorbance increase of 244% (Fig. 4A). However, the aqueous solution, not having the functionalized nanofibrous platform, exhibited an absorbance increase of only 55% (Fig. 4B). A deeper investigation revealed that the aqueous solution had serious background caused by excess free hemin, which was also verified in the corresponding colorimetric reactions (Figure S3A). On the other hand, the functionalized nanofibrous membrane showed a very low detection background signal, possible because free hemin around the membrane was washed off before colorimetric reaction (see Experimental Section). Such low detection background is a key contribution toward enhancing the signal-to-noise ratio. Furthermore, the detection advantage of the functionalized nanofibrous membrane was demonstrated by comparison with the sensing response of a PS thin film. When the PS thin film was treated using the same functionalization process, a very weak color change was observed (Figure S4). Obviously, the large specific surface area of the functionalized nanofibrous membrane efficiently promotes CHA amplification reaction, GOx biocatalysis reaction, and DNAzyme-catalyzed colorimetric reaction. Consequently, the DNAzyme/GOx/PS nanofibrous membrane exhibited an improved performance for the visualization of target HIV.

### Analytical performance of visual biosensor based on the DNAzyme/GOx/PS nanofibrous membrane

**Figure 5:**
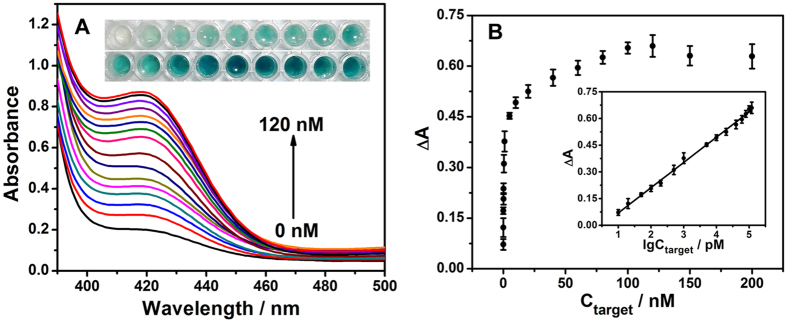
(**A**) UV–vis absorption spectra of colorimetric system for detecting target HIV at various concentrations: 0, 0.01, 0.02, 0.05, 0.1, 0.2, 0.5, 1.0, 5.0, 10, 20, 40, 60, 80, 100, and 120 nM. The inset is the picture of colorimetric system for the detection of target HIV at various concentrations: 0, 0.01, 0.02, 0.05, 0.1, 0.2, 0.5, 1.0, 5.0, 10, 20, 40, 60, 80, 100, 120, 150, and 200 nM. (**B**) The absorbance increase plotted against target HIV concentration. Inset: calibration cure of absorbance increase at 418 nm vs. logarithm of target HIV concentration.

To fabricate a highly sensitive biosensor, some key parameters were optimized including concentrations of avidin, GOx, biotin-H2, hemin and ABTS^2−^, as well as equilibrium time of the nanofibrous membrane in the colorimetric solution, which were 2.0 μM, 0.2 μM, 1.0 μM, 4.0 μM, 2.0 mM, and 20 min, respectively (Figure S5). Under the optimized conditions, the absorbance response was monitored after the nanofibrous membrane was soaked in a series of colorimetric solutions containing target HIV with different concentrations. As shown in Fig. 5A, the absorbance value increased as the concentration of target HIV increased from 0.01 nM to 120 nM. Importantly, its corresponding color gradually changed from colorless to deep green with increasing concentration of target HIV, which is consistent with the absorbance response. The absorbance value was quantified at 418 nm because of the green colored production (ABTS^−^)^10^. A calibration plot of ΔA against lg C was shown in the inset of Fig. 5B. ΔA equates to the absorbance increase at 418 nm after the addition of target HIV, and C is the concentration of target HIV (pM). Each datum in the calibration curve was obtained from an average value of three replicate measurements. All relative standard deviations (RSD) of the measured values were less than 4%. The linear equation of ΔA = −0.072 + 0.14 lg C with a correlation coefficient r of 0.9988. The detection limit was 4.8 pM (S/N = 3), which was better than reported DNA amplification sensors including DNA machine-based fluorescent amplification approach (0.2 nM), CHA amplification-based visual colorimetric biosensor (32 pM) and some nanomaterials amplification strategies^7^,^9^,^31^,^32^,^33^,^34^,^35^,^36^.

### Specificity, reusability and long-term stability of the visual biosensor

**Figure 6:**
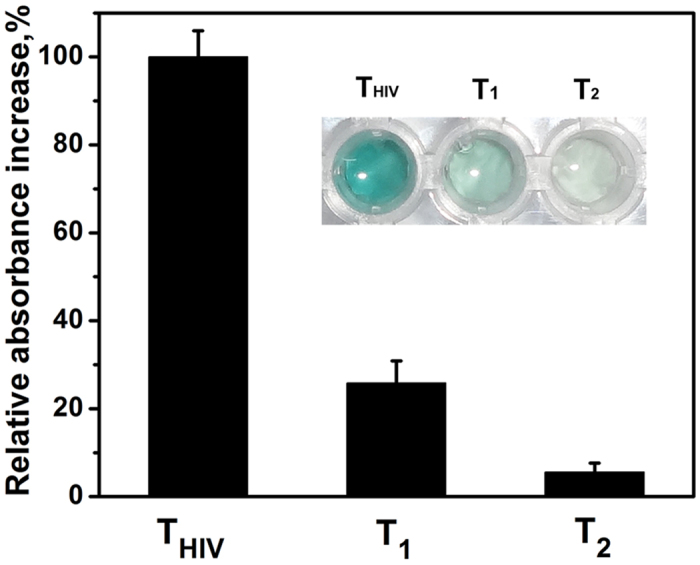
Specificity of the visual biosensor for target HIV (T_HIV_) against single-base (T_1_) and two-base (T_2_) mismatched DNA. The inset is the corresponding photograph of the color change. Concentration of DNA (T_HIV_, T_1_, T_2_) is 5.0 nM.

To evaluate the specificity of the visual biosensor, perfect matched target HIV (T_HIV_) and two similar DNA strands of a single-base (T_1_) and a two-base (T_2_) mismatch were further investigated by comparing the absorbance response of the colorimetric system. Previous reports have demonstrated that mismatched DNA can hybridize with the binding site and, hence, disrupt the G-quadruplex structure^31^,^37^. Consequently, the formation of DNAzyme is inhibited, and catalytic capability for colorimetric reaction is weakened. As shown in Fig. 6, the perfectly matched target HIV produced the highest colorimetric signal at 418 nm. This indicated that the perfect matched DNA triggers the CHA amplification process efficiently. However, in the presence of different mismatched DNA (T_1_ or T_2_), the relative absorbance increase corresponds to 25.9% and 5.6% of the value in the presence of perfect matched target HIV, respectively. The DNAzyme/GOx/PS biosensing platform exhibited a much better discrimination for mismatched DNA in compared with previous reports^31^. Notably, the discrimination at nanomolar level can be visually captured, which could be ascribed to the designing innovation of our multi-amplification nanoplatform.

**Figure 7:**
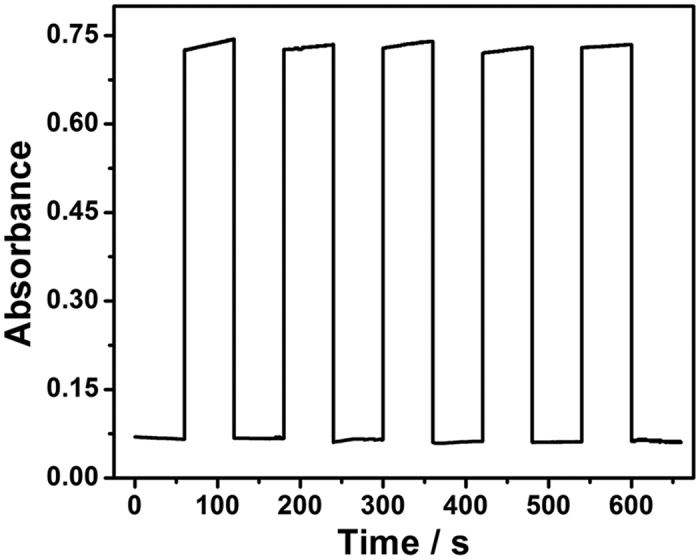
The reusability of the DNAzyme/GOx/PS nanofibrous membrane by separately measuring its absorbance in five cycles of colorimetric system and Tris buffer, respectively. The concentration of target HIV is 100 nM.

The reusability of the nanofibrous membrane was demonstrated by separately measuring the absorbance increase when it was exposed to five cycles of colorimetric system and Tris buffer, respectively (Fig. 7). After the nanofibrous membrane was exposed in the colorimetric solution for 20 min, it turned to deep green. Then, the nanofibrous membrane was moved into Tris buffer until its absorbance value returned to the origin value. Again, when the nanofibrous membrane was soaked in the colorimetric solution for 20 min, its absorbance increase almost recovered to the original value. We repeated the cycle five times using the same DNAzyme/GOx/PS nanofibrous membrane and the RSD of the measured intensities for successive measurements was less than 3%, indicating that the functionalized nanofibrous membrane has a good reusability.

To investigate its long-term stability, the DNAzyme/GOx/PS nanofibrous membrane was stored at 4 °C in a refrigerator, and its absorbance was tested at intervals. Initially, no obvious decrease of absorbance was observed. After storage for 15 days and even one month, the absorbance retained 95.5% and 93.4% of its original value, respectively (Figure S6). The bioactivity of either immobilized GOx or DNAzyme was well maintained, which indicated that the electrospun PS nanofibrous membrane provided a biocompatible microenvironment for biomolecules.

### Target HIV determination in human serum samples

To illustrate the feasibility of the DNAzyme/GOx/PS nanofibrous membrane in a biologically relevant matrix, it was employed to detect the target HIV in real human serum samples. In our work, the human serum samples were simply diluted 100-fold by Tris buffer (pH 7.5) to yield testing sample solutions. In addition, the recovery test was conducted. All data are summarized in Table 1. The results demonstrate that the DNAzyme/GOx/PS nanofibrous membrane offers a reliable method for the determination of target HIV in a biologically relevant matrix.

## Conclusions

A facile, label-free and multi-amplified colorimetric biosensor based on the DNAzyme/GOx/PS nanofibrous membrane was successfully developed for ultrasensitive visualization of HIV DNA biomarkers. By integration of the improved CHA amplification, efficient GOx and DNAzyme biocatalysis on the plasma-treated nanofibrous membrane surface, the proposed DNA biosensor showed high sensitivity, excellent specificity, good reusability and long-term stability. Importantly, single-based mismatch at ultralow concentration in real human serum samples was successfully discriminated. The label-free and multi-amplification nanoplatform provides high potential for visual DNA monitoring at trace levels, which should have wide applications that range from virus detection to genetic disease diagnosis.

## Experimental

### Materials and Reagents

The polystyrene (PS, *M*w ~ 280000), N, N-dimethylformamide (DMF), GOx (153 U/mg), avidin (12.8 U/mg), hemin, dimethyl sulfoxide (DMSO), ABTS, Stains-All, and trizma® hydrochloride (Tris-HCl) were purchased from Sigma-Aldrich (St. Louis, MO, USA). Tetrabutylammonium bromide (TBAB), boric acid, and ethylene diamine tetraacetic acid (EDTA) were purchased from Chengdu Kelong Chemical Factory (Chengdu, China). D-glucose was obtained from Tianjin kemi’ou Chemical Reagent Co. Ltd. (Tianjin, China). Ar gas (purity >99.99%) was purchased from Chengdu Xuyuan Chemical Co., Ltd. (Chengdu, China). Human serum was provided by Nanfang Reagent Factory (Zhejiang, China). All DNA oligonucleotides were synthesized by Shanghai Sangon Biotechnology Co. Ltd. (Shanghai, China). Each hairpin DNA (H1 or biotin-H2) was heated at 95 °C for 2 min and cooled to 25 °C for one hour before use. The hemin stock solution (1 mM) was prepared in DMSO and stored in the dark at −20 °C. Tris buffer solution was prepared by mixing 10 mM Tris-HCl, 5 mM MgCl_2_, 20 mM KCl, and 200 mM NaCl in ultrapure water, and then pH was set at 7.5. The DNA or hemin solution was diluted to the required concentration with Tris buffer. Ultrapure water (18.2 MΩ) was produced from a Millipore system and used throughout the experiments. The sequences of the used oligonucleotides are listed as follows:

H1: 5′-GCT AGA GAT TTT CCA CAC TGA CTT CTC TAG CGG GTT TTG GGT TTT AGT CAG TGT GGA AAA-3′

biotin-H2: 5′-biotin-CTG ACT AAA ACC CAA AAC CCG CTA GAG AAG TCA GTG TGG AAA ATC TCT AGC GGG TTT TGG GTT TTG GGT TTT GGG-3′

Target HIV (T_HIV_): 5′-AGT CAG TGT GGA AAA TCT CTA GC-3′

Target HIV with mismatched base underlined:

T_1_: 5′-AGT CAG TGT GCA AAA TCT CTA GC-3′

T_2_: 5′-AGT CAA TGT GGA AAA ACT CTA GC-3′.

### Apparatus

The size and morphology of PS nanofibrous membrane was recorded by a scanning electron microscope (SEM) (S–4800, Hitachi Ltd., Japan). The absorption spectrum of ABTS^−^ was measured in the wavelength range from 390 to 500 nm using an UV–vis 2900 spectrophotometer (U–2900, Hitachi, Japan). A SONY digital camera (Jiangsu, China) was used to take all the photographs in the experiment. Plasma treatment to PS nanofibrous membrane was processed at atmospheric pressure using low-temperature plasma experimental apparatus (CTP–2000K, Nanjing Suman Electronics Co., Ltd, China) in the presence of argon gas.

### Electrospinning of PS nanofibrous membrane

The electrospinning solution was prepared by dissolving 20 wt% PS and 0.3% (w/v) TBAB into DMF and was stirred with magnetic stirrer at room temperature for 24 h until a transparent solution was obtained. The electrospinning apparatus consisted of a high-voltage power supply (Series EL, Glassman High Voltage Inc.), a syringe pump (74900 series, Cole-Parmer Instrument Company), a stainless steel needle (O.D. = 0.7 mm) and a grounded aluminum foil plate. The electrospinning solution was placed in the syringe and injected through a stainless steel needle with the flow rate of 0.38 mL/h by a syringe pump. The tip-to-collector distance was set about 10 cm. A high voltage of 15 kV was applied to the needle tip, resulting in a continuous jetting stream. The electrospinning process was performed at room temperature with the relative humidity of about 50%. The collection time of the nanofibrous membrane was 2 h. Finally, the collected PS electrospun nanofibrous membrane was dried in an oven at 80 °C for 4 h. A PS nanofibrous membrane was cut into a regular disk-sized pieces (d = 5 mm).

### Fabrication of PS thin film

For the control experiment, one PS thin film contained the same amount of PS as that of an electrospun nanofibrous membrane. In brief, 10 pieces of the PS nanofibrous membrane were completely dissolved with 100 μL of DMF. Then 10 μL of this solution were coated on a clean regular glass disk (d = 5 mm) and dried at room temperature. Finally, the PS thin film was fabricated. The thicknesses of PS nanofibrous membrane and PS thin film were 141.2 ± 7.3 μm and 78.8 ± 6.2 μm, respectively.

### Preparation of DNAzyme/GOx/PS nanofibrous membrane and DNAzyme/GOx/PS thin film

A PS nanofibrous membrane was exposed to argon gas plasmas created by dielectric barrier discharge (DBD) under atmospheric pressure for plasma treatment to modify its surface. First, the PS nanofibrous membrane (d = 5 mm) was attached to the quartz-dielectric reactor (DBD–100A) located at the midpoint of two electrodes to ensure that it would be treated uniformly. Then, argon gas with flow rate of 1.0 L/min was horizontally injected into the plasma system from the side of the quartz-dielectric reactor. The voltage and current was set at 45 V and 2.2 A, respectively, and the treatment time was 2 min. After plasma treatment, the PS nanofibrous membrane was soaked immediately in a 100 μL solution containing avidin and GOx for 1 h. After by soaking in a 100 μL biotin-H2 solution at suitable concentration for 1 h, the membrane was placed into a 100 μL Tris buffer solution containing both H1 and target HIV for 16 h. Finally, it was soaked in a 100 μL hemin solution for 2 h to form the hemin/G-quadruplex DNAzyme. The PS nanofibrous membrane was washed three times with Tris buffer after each soaking, and all experiments were performed at 25 °C. The prepared DNAzyme/GOx/PS nanofibrous membrane was stored at 4 °C. Optimized concentration of avidin (2.0 μM), GOx (0.2 μM), biotin-H2 (1.0 μM), hemin (4.0 μM) and ABTS^2−^ (2.0 mM) and optimized equilibrium time of 20 min were used unless specified otherwise. The functionalization process for the PS thin film was the same as that for the PS nanofibrous membrane.

### Colorimetric and visual biosensing measurement

The colorimetric analysis was performed in colorimetric solution at the room temperature. The colorimetric solution was prepared by mixing 2 mM ABTS^2−^ and 5 mM glucose in the Tris buffer. The DNAzyme/GOx/PS nanofibrous membrane was soaked in 200 μL colorimetric solution. Photographs were taken after colorimetric reaction for 20 min. The UV–vis absorption spectra of colored radical anion ABTS^−^ were measured in the wavelength range from 390 to 500 nm. The absorbance value was recorded at 418 nm. Relative absorbance increase was represented as ΔA = A − A_0_, where A is the absorbance value of the colorimetric system with target HIV and A_0_ is the absorbance value without target HIV.

### Native polyacrylamide gel electrophoresis

Native polyacrylamide gel (12%) was prepared with 1× TBE buffer (89 mM Tris, 89 mM boric acid, 2 mM EDTA, pH 8.3). 10 μL of each sample was mixed with 2 μL of 6× gel loading buffer (0.25% bromophenol blue, 0.25% xylene cyanol, 40% (w/v) sucrose solution) before loading into the gels. The gels were run under a constant voltage of 110 V over a period of about 1 h. Then the gels were stained using Stains-All to image the position of DNA and photographed by a digital camera.

## Additional Information

**How to cite this article**: Long, Y. *et al*. Ultrasensitive Visual Detection of HIV DNA Biomarkers via a Multi-amplification Nanoplatform. *Sci. Rep*. **6**, 23949; doi: 10.1038/srep23949 (2016).

## Supplementary Material

Supplementary Information

## Figures and Tables

**Table 1:** Determination and recovery of target HIV in human serum samples using the DNAzyme/GOx/PS nanofibrous membrane.

Samples	DNA added (nM)	DNA found (nM)	Recovery (%)	RSD^d^ (%)
T_HIV_^a^	5.0	4.8	96	3.5
T_1_^b^	5.0	4.7	94	1.7
T_2_^c^	5.0	4.7	94	1.9

^a^The perfect complementary target HIV.

^b^The single-base mismatched target HIV.

^c^The two-base mismatched target HIV.

^d^Three replicates were performed.
