# Supplementary material for: Ultrasensitive Visual Detection of HIV DNA Biomarkers via a Multi-amplification Nanoplatform
Source: Sci Rep. 2016 Apr 1;6:23949. doi: 10.1038/srep23949 (PMC4817037; doi:10.1038/srep23949)
Supplement: Supplementary Information [file srep23949-s1.pdf]

Supporting Information

Ultrasensitive Visual Detection of HIV DNA Biomarkers via a Multi-amplification Nanoplatform

Yuyin Long[1], Cuisong Zhou[1,], Congmin Wang[1], Honglian Cai[1], Cuiyun Yin[1], Qiufang Yang[1], Dan Xiao [1,2,*]*

[1]College of Chemistry, Sichuan University, 29 Wangjiang Road, Chengdu 610064, People's Republic of China

[2]College of Chemical Engineering, Sichuan University, 29 Wangjiang Road, Chengdu 610065, People's Republic of China

***** To whom correspondence should be addressed

e-mail: zcs@scu.edu.cn (C. S. Zhou)

e-mail: xiaodan@scu.edu.cn

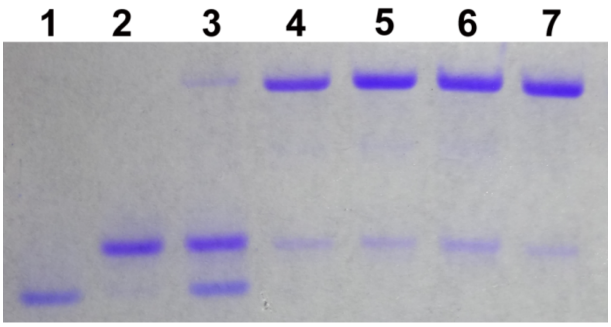

Supplementary Figure S1. Native PAGE analysis of the CHA amplification reaction. Lane 1: H1; Lane 2: H2; Lane 3: H1 + H2; Lane 4: H1 + H2 + 100 nM T_{HIV}; Lane 5: H1 + H2 + 200 nM T_{HIV}; Lane 6: H1 + H2 + 500 nM T_{HIV}; Lane 7: annealed (H1 + H2). [H1] = [H2] = 1 μM.

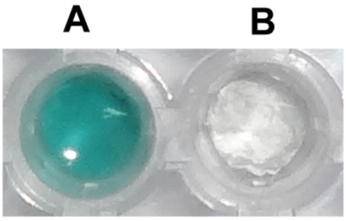

Supplementary Figure S2. Color change of functionalized PS nanofibrous membrane with **(A)** and without **(B)** plasma treatment in the colorimetric solution. The concentration of target HIV is 100 nM.

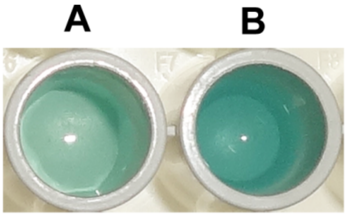

Supplementary Figure S3. Color change of aqueous solution without **(A)** and with **(B)** 5.0 nM HIV DNA in the colorimetric system.

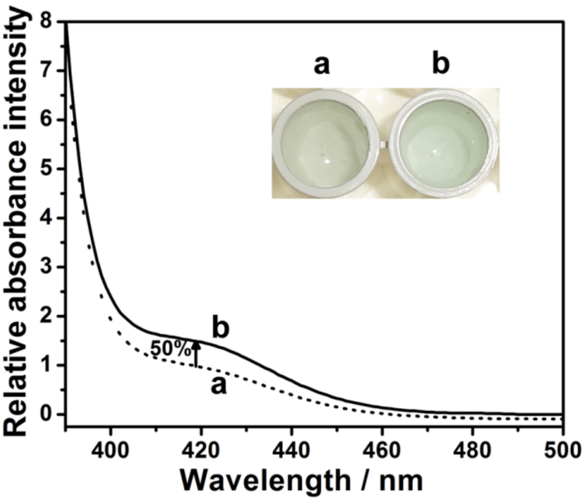

Supplementary Figure S4. Performance of the DNAzyme/GOx/PS thin film without **(a)** or with **(b)** 5.0 nM HIV DNA in the colorimetric system. The inset shows corresponding photograph of the color change.

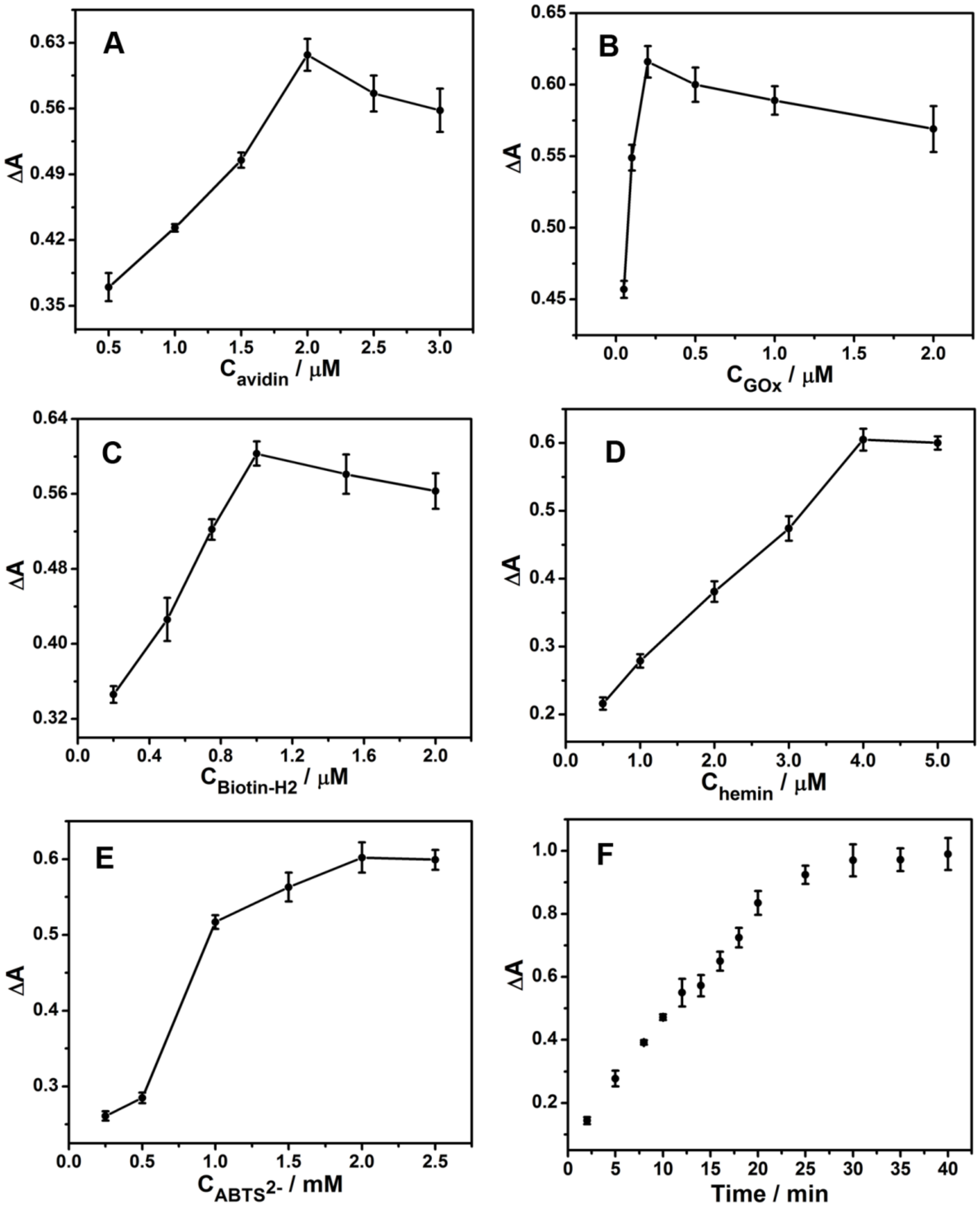

Supplementary Figure S5. Effects of various conditions on the absorption response of the colorimetric system. Concentration of **(A)** avidin, **(B)** GOx, **(C)** biotin-H2, **(D)** hemin, **(E)** $ABTS^{2-}$, and **(F)** equilibrium time of DNAzyme/GOx/PS nanofibrous membrane soaked in colorimetric solution.

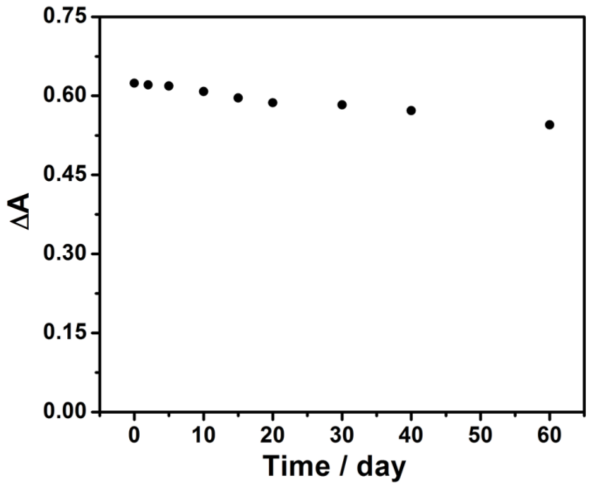

Supplementary Figure S6. Stability of the DNAzyme/GOx/PS nanofibrous membrane in Tris buffer (pH = 7.5). The concentration of target HIV is 100 nM.
